# Induction of Oligodendrocyte Differentiation and In Vitro Myelination by Inhibition of Rho-Associated Kinase

**DOI:** 10.1177/1759091414538134

**Published:** 2014-06-18

**Authors:** Carlos E. Pedraza, Christopher Taylor, Albertina Pereira, Michelle Seng, Chui-Se Tham, Michal Izrael, Michael Webb

**Affiliations:** 1 EMD Serono Research & Development Institute, Inc., Billerica, MA, USA; 2 Kadimastem, Ness Ziona, Israel

**Keywords:** cytoskeletal dynamics, human OPCs, maturation, myelin regeneration, oligodendrocytes, ROCK inhibition

## Abstract

In inflammatory demyelinating diseases such as multiple sclerosis (MS), myelin degradation results in loss of axonal function and eventual axonal degeneration. Differentiation of resident oligodendrocyte precursor cells (OPCs) leading to remyelination of denuded axons occurs regularly in early stages of MS but halts as the pathology transitions into progressive MS. Pharmacological potentiation of endogenous OPC maturation and remyelination is now recognized as a promising therapeutic approach for MS. In this study, we analyzed the effects of modulating the Rho-A/Rho-associated kinase (ROCK) signaling pathway, by the use of selective inhibitors of ROCK, on the transformation of OPCs into mature, myelinating oligodendrocytes. Here we demonstrate, with the use of cellular cultures from rodent and human origin, that ROCK inhibition in OPCs results in a significant generation of branches and cell processes in early differentiation stages, followed by accelerated production of myelin protein as an indication of advanced maturation. Furthermore, inhibition of ROCK enhanced myelin formation in cocultures of human OPCs and neurons and remyelination in rat cerebellar tissue explants previously demyelinated with lysolecithin. Our findings indicate that by direct inhibition of this signaling molecule, the OPC differentiation program is activated resulting in morphological and functional cell maturation, myelin formation, and regeneration. Altogether, we show evidence of modulation of the Rho-A/ROCK signaling pathway as a viable target for the induction of remyelination in demyelinating pathologies.

## Introduction

Multiple sclerosis (MS) is characterized by a progressive degeneration of axons and the neurons from which they arise. Although the mechanisms of neuronal degeneration are likely multifactorial, the proximal cause of the vulnerability of axons that leads to their eventual loss is their demyelination as a result of an inflammatory environment, a process long recognized as the hallmark of MS. All therapies currently approved for MS are focused on control of the inflammatory process. While these therapies reduce relapse rate during relapsing–remitting MS (RRMS), they do not prevent disease evolution to the progressive form, and they become ineffective once this transition has occurred. Currently, there is a significant body of opinion that therapies aimed at prevention of disease progression and restoration of function will include mechanisms protecting neurons and repairing demyelination.

The central nervous system (CNS) has a substantial ability to remyelinate axons, which can be clearly distinguished from primarily myelinated axons in electron micrographs of MS plaques (Bruck et al., 1997; Bruck, Kuhlmann, & Stadelmann, 2003; Erickson, 2008; Staugaitis, Chang, & Trapp, 2012). This secondary myelination is a result of maturation of endogenous oligodendrocyte precursor cells (OPCs), which persist throughout life and constitute a substantial proportion of cells in the CNS (∼4%; Keirstead & Blakemore, 1999; Keough & Yong, 2013; Staugaitis et al., 2012). For reasons that are not fully understood, this remyelination capacity is lost in later phases of MS (Hagemeier, Bruck, & Kuhlmann, 2012; Hanafy & Sloane, 2011). This is not due to depletion of the OPC pool, as these cells can be readily identified even in long-term patients and within lesions (Chang, Nishiyama, Peterson, Prineas, & Trapp, 2000; Kipp, Victor, Martino, & Franklin, 2012; Wolswijk, 2002). However, their ability to differentiate and remyelinate appears to be blocked. If this block could be overcome pharmacologically, this might result in restoration of remyelination capacity, a long-lasting protection of axons, and a restoration of their function even in progressive MS.

In the initial phases of OPC maturation, early bipolar cells extend processes to seek axons for myelination. They form lamelipodia, by aligning microtubules in a wide, barbed-end structure and subsequently transport actin microfibers to the leading edge to protrude forward, forming filopodia and elongated extensions (Song, Goetz, Baas, & Duncan, 2001). In this phase, regulation of cytoskeletal dynamics is the most prominent feature (Song et al., 2001; Wang, Tewari, Einheber, Salzer, & Melendez-Vasquez, 2008; Wang et al., 2012). Contact with an appropriate axon substrate results in triggering a further program of gene expression in which the immature oligodendrocyte wraps processes around the target axon and synthesizes large amounts of specialized myelin lipids and proteins.

Thus, the initial phase of OPC differentiation depends strongly on the correct spatial and temporal polymerization of tubulin and actin, and the assembly of activated nonmuscle myosin with actin fibers (Mullins, Heuser, & Pollard, 1998; Ridley et al., 2003; Svitkina & Borisy, 1999; Svitkina et al., 2003). Among the multiple proteins involved in controlling cytoskeletal alterations in general and microfilament, microtubule stabilization in particular, the Rho-guanosin triphosphatase (GTPase)/Rho-associated kinase (Rho/ROCK) pathway is notable not only for its role in regulating these functions but also for its accessibility as a source of druggable targets (Chang, Bean, & Jakobi, 2009; Hahmann & Schroeter, 2010; Henderson et al., 2010; Olson, 2008). ROCK (Ser/Thr. EC 2.7.11.1) is activated by binding of Rho-GTP (active) to its Rho-binding domain, which induces a conformational change in the enzyme exposing its kinase domain and rendering it active (Ridley, 2001; Schofield & Bernard, 2013; Somlyo & Somlyo, 2000). Activated ROCK phosphorylates myosin phosphatase subunit one (MYPT1), thereby inactivating it, and thus indirectly increasing myosin light chain (MLC) phosphorylation levels, which enables contraction of stress fibers (Velasco, Armstrong, Morrice, Frame, & Cohen, 2002). Activated ROCK also affects microfilament stabilization through phosphorylation of its downstream target LIM-kinase (LIMK), which in turn deactivates the microfilament severing protein cofilin, thus favoring the formation of stress fibers (Amano, Tanabe, Eto, Narumiya, & Mizuno, 2001; Bernard, 2007; Yang et al., 1998).

Previous studies using statins have implicated the Rho/ROCK pathway in promoting oligodendrocyte differentiation and myelination in experimental allergic encephalomyelitis (EAE) studies (Miron et al., 2007; Paintlia et al., 2005; Paintlia, Paintlia, Singh, & Singh, 2008). The effects of statins are complex, but part of their action is mediated through the Rho kinase pathway by inhibition of the isoprenylation required by Rho GTPases for their translocation to membranes and activation and interaction with downstream effectors. The treatment of cultured human OPC with simvastatin (Miron et al., 2007) resulted in a biphasic effect, with differentiation followed by process retraction and death. The former could be mimicked by the commercially available Rho kinase inhibitors H1152 and Y-27632.

In this study, we investigated further the effect of ROCK inhibition on oligodendrocyte differentiation by the use of morphological, myelin protein expression and target inhibition analyses in cells generated from both rodent and human sources. We also tested the induction of myelin formation in a coculture system on ROCK inhibition and evaluated the viability of this kinase as a potential target for remyelination in MS.

## Materials and Methods

### Neurosphere-Derived OPCs

Neurospheres (Nph) were prepared from embryos at 14.5 days of gestation obtained from timed pregnant females of C57/Bl6 mice, as previously described (Kornblum, Yanni, Easterday, & Seroogy, 2000; Pedraza, Monk, Lei, Hao, & Macklin, 2008). In brief, after separation of meninges and cerebellum, cerebrum tissue was mechanically triturated with a 1-ml micropipette until total tissue dissociation was achieved, filtered through a 70 -µm cell strainer (Fisher Scientific, Pittsburgh, PA), and plated in 25-cm^2^ plastic culture flasks (2 brains/flask). Nph proliferation media (NPM) consisted of Dulbecco’s Modified Eagle Medium (DMEM)/F12, B27 neuronal supplement (Gibco, Baltimore, MD) and 10 ng/ml endothelial growth factor (EGF; Sigma, St. Louis, MO). After 48 to 72 hr, floating Nphs were passaged at a 1 : 1 ratio into 75-cm^2^ culture flasks. After a further 48 to 72 hr, they were passaged into 150-cm^2^ flasks. Subsequent passages were performed every 3 to 4 days at a ratio of 1 : 2 or 1 : 3 in the same media, and Nph were used to generate OPCs for no more than 12 passages.

OPCs were generated by chemical disaggregation of Nph with a mouse neural progenitor dissociation kit following the manufacturer instructions (NeuroCult, StemCell Technologies, AB, Canada). The single-cell suspension was then filtered through a 40 -µm cell strainer and plated on poly-d-lysine (PDL)-coated plates. Cells were maintained in oligodendrocyte proliferation media (OPM) consisting of DMEM/F12, B27 neuronal supplement, and 10 ng/ml platelet-derived growth factor (PDGF)/fibroblast growth factor-2 (bFGF; Peprotech, Rocky Hill, NJ). Bipolar cells were observed shortly after plating and proliferated rapidly when maintained in this medium. The oligodendrocyte cell lineage of these cells has been demonstrated previously (Pedraza et al., 2008).

### Mixed Glia-Derived Rat OPCs

Rat postnatal OPCs were obtained as described elsewhere according to the original procedure of McCarthy and DeVellis (1980). In brief, postnatal Day 1 rat pups were sacrificed, and cerebral cortices dissected and mechanically dissociated in medium composed of DMEM, 10% heat inactivated fetal bovine serum, and 5 µg/ml penicillin/streptomycin. Dissociated cells were plated and maintained in 75-cm^2^ culture flasks for 10 days, replenishing with fresh media at Days 3 and 7. At Day 10, the flasks were shaken on a heated (37℃) shaker for 1 hr at 110 rpm to separate loosely attached microglial cells. The medium was replenished, and the cells incubated for the remainder of the day. To dislodge OPCs from the astrocyte monolayer, flasks were shaken overnight (210 rpm, 37℃). The medium was collected and centrifuged, and cell pellets were resuspended in medium consisting of DMEM/F12, B27 neuronal supplement, and 10 ng/ml PDGF/bFGF. Cells were then plated on PDL-coated tissue culture plastic and treated with test compounds.

### Cerebellar Explants

Myelination–demyelination–remyelination studies in mouse cerebellar organotypic cultures were carried out according to a protocol for rat tissue explants (Birgbauer, Rao, & Webb, 2004), with minor modifications. In brief, after careful dissection to keep the tissue intact, cerebella of postnatal Days 4 to7 wild-type mouse pups were sectioned at 400 -µm thickness using a Leica VT1000S vibratome. Cerebellar sections were then plated on insert plates (Corning, Wilkes Barre, PA), and a few droplets of high-glucose medium (50% DMEM/F12, 25% Hank’s buffered salt solution [Gibco], 25% horse serum, 5 mg/ml glucose, and penicillin/streptomycin [Gibco]) were added to cover the tissue during the first 6 hr to allow attachment of the slices to the insert plate surface. When the slices were firmly attached, fresh medium was added to cover the tissue, and the cultures were maintained under these conditions for 4 days. To induce demyelination, 0.5 mg/ml lysolecithin (l′-monoacyl-l-3-glycerylphosphorylcholine; Sigma) was added to the culture medium for 17 hr, after which it was removed and replaced with fresh medium. Treatments with compounds were initiated at this point, and the cultures maintained for 4 to 10 additional days. Demyelination–remyelination was assessed by real-time polymerase chain reaction (PCR).

### Immunocytochemistry

OPCs were fixed with 4% paraformaldehyde in phosphate-buffered saline (PBS) for 10 min at room temperature (RT), washed three times with PBS, and permeabilized with 0.1% Triton X100 for 10 min at RT. After blocking with 3% bovine serum albumin (BSA) in PBS for 60 min at RT, the cells were incubated with primary antibodies at the indicated concentrations overnight at 4℃. The cells were washed three times with PBS incubated with secondary antibodies for 45 min at RT and washed again before being visualized by fluorescence microscopy. Primary antibodies used in this study were mouse anti-β-III-tubulin (1 : 300; Chemicon, Temecula, CA), rabbit anti-myelin basic protein (MBP, 1 : 300; EMD Millipore, Billerica, MA), and anti-rabbit or anti-mouse AlexaFluor secondary antibodies (Life Technologies, Grand Island, NY) used at a dilution of 1 : 700 in PBS.

### Human Stem Cell-Derived Oligodendrocyte Progenitor Cell Cultures and Cocultures with Rat Dorsal Root Ganglion Neurons

Human oligodendrocytes were generated by a proprietary methodology of Kadimastem (Weizmann Science Park, Nes-Ziona, Israel; www.kadimastem.com). In brief, 5,000 human embryonic stem cell-derived OPCs (hES-OPCs) were seeded in each well of Matrigel and PDL-coated 96-well black μClear plates (Greiner, Frickenhausen, Germany). The cells were seeded in 100 μl of differentiation media (DIF) in each well. Twenty-four hours after seeding, medium was removed, and 200 μl of DIF fresh medium containing the compound of interest at a specific dilution (in 0.1% DMSO) was supplemented to the cells (first feeding). Total volume for each well was 200 μl. Four replicates were used for each dose of compound under study. After 48 hr, the media were replenished by removing 100 μl of media, and the same amount of fresh DIF media was added to the wells. Only at the first feeding, DIF media with 0.1% DMSO were used as control of the compound in medium containing 0.1% DMSO. Two experimental sets were conducted for the differentiation assay. On Day 21, live staining of oligodendrocytes was done by incubating the cells with O4 antibody (R&D Systems, Minneapolis, MN) for 20 min followed by detection with goat anti-mouse IgM-FITC (Chemicon) for additional 20 min. Cells were washed twice using DIF media. Immediately after, cells were fixed using 4% paraformaldehyde, and then washed with 1 × PBS, and 4′,6-diamidino-2-phenylindole (DAPI; Sigma) was used for nuclear staining.

The ArrayScan VTI HCS reader system (Cellomics, Thermo-scientific) was used for scanning 20 fields for each tested well under 10× objective. Cellomics Neural profiling bioapplication was used for processing and analyzing the images. The 4-parameter Hill-Slope curve fittings were generated by GraphPad Prism software using the least squares fitting method, and EC_50_ values were calculated for the three major parameters of oligodendrocyte differentiation: number of differentiated oligodendrocytes, number of oligodendrocyte processes, and oligodendrocytes processes length (µm). The negative control groups of each experimental set were used for data normalization.

Human pluripotent stem cell-derived OPC (hSC-OPC)/recombinant dorsal root ganglion (rDRG) cocultures were carried out as follows: 5,000 hES-OPCs were seeded on top of 14-day-old DRG neuron axons in each well of 96-well black μClear plates (Greiner). The cells were seeded in 100 μl of myelination media (MYL) for each well. Twenty-four hours after seeding, media were gently removed, and a fresh MYL medium that contained the compound of interest at a specific dilution (in 0.1% DMSO) was supplemented to the media (first feeding), and the total volume for each well was 200 μl. For each tested compound dose, four replicates were used. After 48 hr, the media were replenished by removing 100 μl of media, and fresh MYL medium (that contains 0.1% DMSO) was added to the wells. For all wells, MYL media was changed every other day by removing 100 μl of media, and 100 μl of fresh MYL media (with 0.1% DMSO) was added to each well. Two experimental sets were conducted for myelination assay. On Day 28, cells were fixed using 4% paraformaldehyde, washed twice with 1× PBS, and kept at 4℃ before staining. Nonspecific staining was blocked with normal goat serum (5% w/v in PBS), and 0.3% Triton (TX-100) was used for cell permeabilization for 30 min at RT. Cells were washed twice with 1× PBS. For oligodendrocyte myelin staining, the cells were incubated with rat anti-MBP (Abcam, Cambridge, MA) for 1 hr and washed twice with 1× PBS. Then the cells were incubated with goat anti-rat IgG-Cy3 (Jackson Lab) for 30 min and washed twice. For staining neural axons, cells were incubated with rabbit polyclonal anti-neurofilament (NF; Abcam) for 1 hr and then washed twice with 1× PBS. Then, cells were incubated with Alexa Fluor® 488 goat anti-rabbit (Invitrogen) for 30 min and washed twice with 1× PBS. Following MBP and NF, DAPI staining (Sigma) was used for nuclear staining.

The ArrayScan VTI HCS reader system (Cellomis, Thermo-scientific) was used for scanning 49 fields for each tested well under 10× objective. A modified version of Cellomics Neural profiling and colocalization bioapplications was developed for analyzing human oligodendrocyte myelination of DRG neuron axons. The four-parameter Hill-Slope curve fittings were generated by GraphPad Prism software using least squares fitting method, and EC_50_ values were calculated for the three major parameters of oligodendrocyte myelination: number of differentiated oligodendrocytes, areas of myelinated axons in pixels (MBP and NF overlap area), and number of myelinated regions (MBP and NF overlap count). The negative and positive control groups of each experimental set were used for data normalization.

### RNA Extraction and Real-Time PCR (Quantitative RT-PCR)

Total RNA was isolated from single-cell cultures or tissue explants using Tri-Zol reagent (Gibco). cDNA was generated from 1 to 3 µg of total RNA by Retrotranscriptase II (Gibco) reaction, and the selected transcripts were detected and amplified by quantitative RT-PCR in an Applied Biosystems (Grand Island, NY) 7500 fast qPCR machine, following the manufacturer’s instructions. The results are presented as specific mRNA expression relative to either β-actin or β-tubulin, used as internal controls as indicated.

### ROCK Expression Knockdown by Small Interfering RNA

Nph-OPCs (5.5 × 10^6^) were plated on PDL-coated 100-mm^2^ plates for 24 hr in OPM. The cells were then transfected with a final concentration of 30 nM small interfering RNA (siRNA) pools against ROCKI or ROCKII or both (Dharmacon, Thermofisher, Pittsburgh, PA). This system pools four siRNA-validated sequences against a specific target achieving ∼70% of mRNA knockdown according to the manufacturer’s brochure. Transfection reagent used was Dharmafect following the product’s instructions, and the experiments were performed three times in duplicate plates. Catalog numbers for ROCKI, ROCKII, and nontargeting control were L-046504-00-0005, L-040429-00-0005, and D-001810-10-055, respectively. Six hours after transfection, OPM was changed and cell lysates for western blotting or Trizol extracts for total RNA isolation were made 72 hr later.

### Sodium Dodecyl Sulfate–Polyacrylamide Gel Electrophoresis and Western Blot

Tris-glycine acrylamide-bysacrylamide gradient gel electrophoresis was performed as follows: After washing the cells with ice-cold PBS, protein lysates were made by adding 50 µl of PhosphoSafe extraction reagent (EMD Millipore) supplemented with antiprotease cocktail (cOmplete; Roche, Branford, CT) onto each 100-mm^2^ cell culture plate. After detaching the cells with the help of a cell scraper and disrupting cell membranes by sonication, the protein extracts were centrifuged (10,000 *g*, 10 min, 4℃) and the supernatants separated. Protein content was measured by the BCA protein determination method (Biorad, Hercules, CA), and 30 µg of total protein was mixed with SDS sample buffer (Laemmli) supplemented with 1% β-mercaptoethanol. After heating the samples to 70℃ for 10 min, they were loaded onto 4% to 20% acryl-bysacrylamide gradient gels, and electrophoresis was carried out at 120 volts with constant amperage.

Resolved proteins were then transferred onto nitrocellulose membranes using the iBlot methodology and device (Life technologies). Unspecific binding was blocked with odyssey blocking buffer (Li-Cor Biosciences, Lincoln, NE; 1 hr, RT) and incubated with the following primary antibodies: rabbit anti-MBP, mouse anti-proteolipid protein (PLP), mouse anti-actin, and mouse anti-glyceraldehyde 3-phosphate dehydrogenase antibodies were all from EMD-Millipore (AB980, MAB388, MAB1501, and MAB347, respectively); rabbit anti-ROCKI antibody and rabbit anti-ROCKII antibody were from Proteintech (Chicago, IL; 20248-1 and 20248-2, respectively); and rabbit antiphosphorylated MYPT1 (ser507) and rabbit anti-MYPT1 were from Cell Signaling (Danvers, MA; 3004 and 2634, respectively). Secondary detection was performed using Li-Cor IRDye secondary anti-mouse and anti-rabbit antibodies, and near-infrared signal was detected by Li-Cor’s Odyssey imaging system.

### Statistical Analysis

For statistical analysis, paired Student *t* test was conducted. The results are shown as mean ± SEM and mean ± SD as indicated.

## Results

### Morphological Analysis of Mouse Oligodendrocyte Progenitors

**Figure 1.**
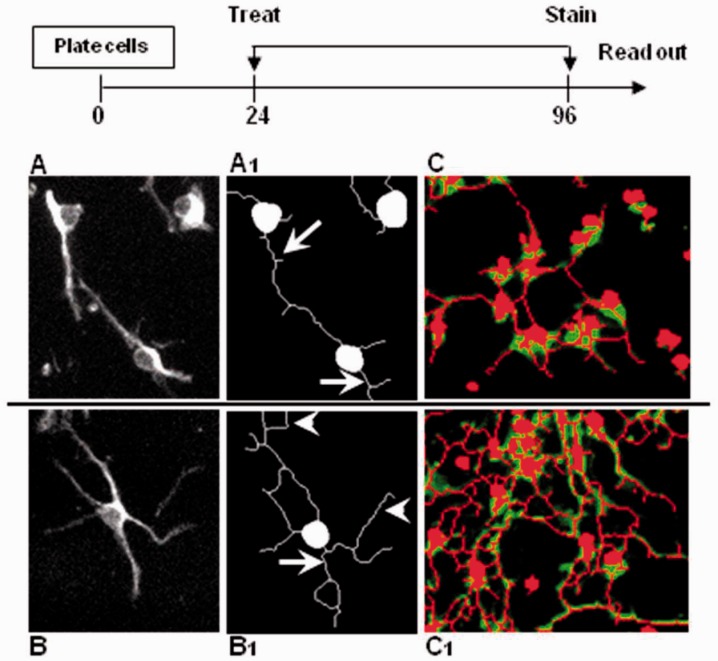
Morphology-based oligodendrocyte precursor differentiation assay. Mouse neurosphere-derived oligodendrocyte precursor cells (Nph-OPCs) transform from bipolar, fusiform cells (a, a1) to multiprocessed, branched, differentiated OPCs (b, b1) in response to maturation stimuli. Morphological changes in OPCs were measured with high-content image software that allows quantification of thick processes (a1, arrows) and derived branches (b1, arrowheads) associated to a cell body. In this paradigm, the cells were treated with compounds after 24 hr of plating for an additional 72 hr when membrane staining, image acquisition, and outgrowth analysis were performed (top diagram); c and c1 show over-imposed, digitally generated cell bodies and processes in bipolar and branched cells.

**Figure 2.**
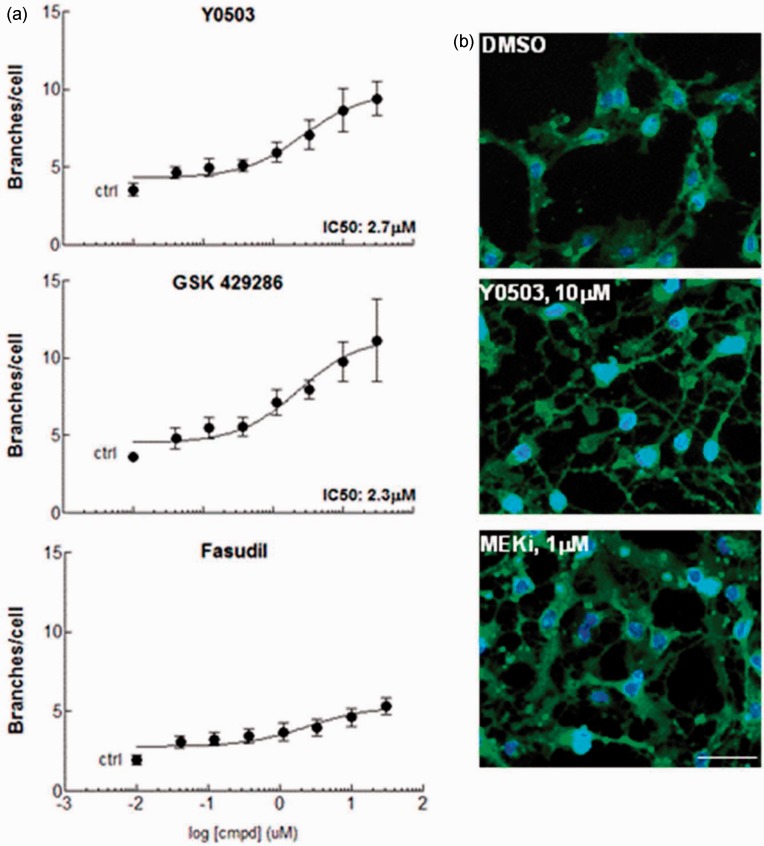
Rho-kinase inhibitors induce oligodendrocyte precursor cell (OPC) differentiation. Treatment of mNph-OPC with the Rho-associated kinase (ROCK) inhibitors Y0503, GSK 429286, and Fasudil results in the generation of processes and branches in a dose-response manner. Compounds with high inhibitory potency in enzymatic assays (Table 1) induced measurable IC_50_ values in the OPC differentiation assay in the low micromolar range as compared with Fasudil, which showed a minor differentiation effect (a). OPC differentiation induced by inhibition of ROCK was characterized by the extension of long, thin processes and multiple branches (b, Y0503), while other maturation stimulus like inhibition of the mitogen-activated protein kinase (MEK), resulted in thick processes and membranous structures in the same treatment period (b, MEKi). Cells were visualized after membrane staining with Calcein-AM (b). All concentration response experiments were performed in triplicate, at least three times using different culture preparations. Images used for neurite outgrowth quantification were taken at low magnification accounting for a range of 550 to 1,250 cells/image/well.

To determine the effects of ROCK inhibition on OPCs, we developed a high-content image morphological analysis of OPC differentiation using mouse embryonic neural progenitor-derived OPCs (Nph-OPCs). Neural progenitors grow as nonadherent spheroids known as neurospheres (Nph; Chojnacki & Weiss, 2008; Tropepe et al., 1999), which are considered to be a mixed population of neural stem cells and progenitors of the CNS (Rao, 1999; Zhou & Chiang, 1998). These cells have been previously used in multiple published studies for the generation of high-yield, high-purity cultures of OPCs from rodents (Calaora et al., 2001; Chojnacki & Weiss, 2004; Lachyankar et al., 1997; Pedraza et al., 2008). After 24 hr of plating on PDL-coated tissue culture plastic, Nph-OPCs were treated as indicated with differentiation stimuli and further incubated for 72 hr. At this time, a solution (4 µl) containing final concentrations in the well of 1 µM Calcein-AM and 100 nM Hoechst (Invitrogen) was added to the wells for 30-min incubation prior to image capture. Four images per well were analyzed using a neurite outgrowth module (Molecular Devices, Sunnyvale, CA). We quantified the number of branches and total outgrowth per cell (Figure 1). This methodology permitted the accurate detection of fine branches extending from thick processes (branch analysis) and determination of the total number of cellular extensions generated by individual cells and all the cells in the images (total outgrowth). Additionally, nuclear staining by Hoechst allowed the accurate association of branches and processes to their corresponding cell bodies and permitted quantification of total cell numbers in each well. Using this technique, dose-response curves and IC_50_ values were consistent and reproducible across several replicates and between experiments (Figures 1 and 2).

### Nph-OPC Differentiation Induced by ROCK Inhibitors

Using an automated version of the digital morphological assay described earlier, we tested the effect of several commercially available ROCK inhibitors (ROCKi; Figure 2 and Table 1) on the differentiation of mouse Nph OPC. These inhibitors included Fasudil, a ROCK inhibitor in use in Japan for more than 15 years for the treatment of brain vasospasm (Hirooka & Shimokawa, 2005), and two other small molecules widely used in ROCK basic research in diverse areas: Y-27632 (trans-4-[(1 R)-1-aminoethyl]-*N*-4-pyridinylcyclohexanecarboxamide dihydrochloride; Koyanagi et al., 2008; Narumiya et al., 2000) and GSK 429286: 4-[4-(trifluoromethyl)phenyl]-*N*-(6-fluoro-1H-indazol-5-yl)-2-methyl-6-oxo-1,4,5,6-tetrahydro-3-pyridinecarboxamide; Goodman et al., 2007; Nichols et al., 2009). Generation of branches was measured in dose-response experiments yielding IC_50_ values of ∼2.5 to 2.9 µM for these compounds (Figure 2(a)). OPC differentiation induced by ROCKi comprised the formation of a few long, thick processes and extremely ramified branched networks (Figure 2(b)). This effect was in contrast to the effect of other differentiation stimulus such as inhibition of the mitogen-activated protein kinase, which induced a *blossom-like* morphology with thick processes and interdigital membranous structures (Figure 2(b)). Branching induced by potent and specific inhibitors such as Y27632, also known as Y0503, and GSK 429286 was more extensive than that induced by the relatively weaker inhibitor Fasudil (Narumiya et al., 2000; Figure 2(a)). Importantly, we did not observe a significant increase in cell proliferation in response to ROCKi as measured by the number of Hoechst-labeled nuclei across different conditions (DMSO: 522 ± 126; Fasudil 1 µM: 598 ± 21; Y0503 1 µM: 625 ± 88 cells/well, *n* = 3 in triplicate wells counting cells from three different Nph preparations).

**Table 1.** ROCK Inhibitors Used in This Study.

Compound	Activity, IC_50 _µM
Y0503	ROCKI: 53
	ROCKII: 48
	NCBI: 123862
	(Narumiya, Ishizaki, & Uehata, 2000)
GSK 429286	ROCKI: 14
	ROCKII: 48
	NCBI: 11373846
	(Goodman et al., 2007; Nichols et al., 2009)
Fasudil	ROCKI: 370
	ROCKII: 170
	NCBI: 163751
	(Sward et al., 2000)

*Note.* ROCK = Rho-associated kinase.

### Interspecies Effect of ROCK Inhibition on OPC Differentiation

To assess the effect of ROCK inhibition in cultured OPCs representing different stages of differentiation and species, we performed, in addition to mouse Nph-OPC, analyses of OPC differentiation in cells obtained from rat mixed glial cultures and in OPCs derived from human stem cells.

**Figure 3.**
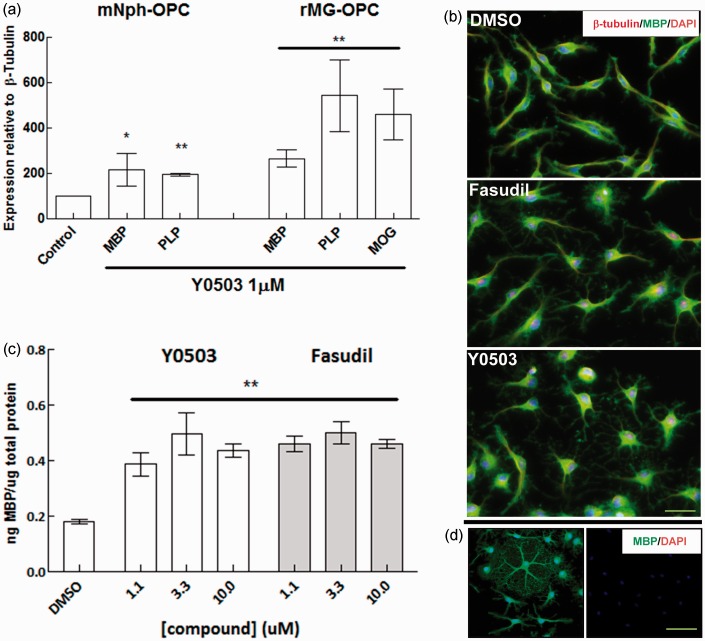
Induction of myelin protein expression in response to Rho-associated kinase (ROCK) inhibition. Accelerated oligodendrocyte precursor cell (OPC) differentiation in response to ROCK inhibition was observed at the gene expression level as myelin basic protein (MBP) and proteolipid protein (PLP) mRNA increased significantly in mouse- and rat-derived OPCs exposed to Y0503 for 4 days (a). Increased expression of myelin oligodendrocyte glycoprotein (MOG) was observed only in rat cells, indicating a more advanced stage of these cells at the moment of treatment. Rat OPCs showed positive immunostaining for MBP (b, green) after 3 days in vitro, which was increased and localized on extending branches and processes in cells treated with ROCK inhibitors Fasudil and Y0503 (b). β-Tubulin coimmunostaining (b, red) helps visualize thin processes and larger cell extensions. MBP-positive immunostaining (d, left image) was verified by the use of primary isotype (IgG) antibody control (d, right image). MBP protein synthesis was consequently increased in rat OPCs treated twice with both Y0503 and Fasudil in a 5-day period after three previous days of plating as determined by ELISA (c). Bar: 50 µm. **p < 0.01; *p < 0.05 according to Student *t* test.

OPCs isolated from rat mixed glial cultures are known to mature readily in the absence of mitogens achieving a premyelinating OL stage as determined by the expression of the cell surface sulfatide O4, and the myelin proteins MBP and PLP among others (Armstrong, 1998; Barbarese & Pfeiffer, 1981; Cammer, 1999; Jung-Testas, Schumacher, Robel, & Baulieu, 1996; Konola, Tyler, Yamamura, & Lees, 1991). They also display marked morphological changes, progressing from bipolar to multibranched cells generating membranous structures, and probably represent a later developmental stage than Nph-OPC. In rat mixed glial OPCs, we measured myelin protein expression at the RNA level by qPCR and MBP production by ELISA (MBL International, Woburn, MA). In short-term treatments with ROCKi (48–72 hr), morphological changes consistent with early stage differentiation (increased cell processes and branches) were clearly seen using immunocytochemistry for MBP and β-tubulin (Figure 3(b)), but we did not observe a significant increase in myelin protein expression. However, in long-term exposure experiments (3 days maturation +4 days treatment) including renewal of the stimulus, both Fasudil and Y0503 induced a significant increase in the expression of MBP, PLP, and myelin oligodendrocyte glycoprotein (MOG) mRNAs at concentrations as low as 0.1 µM (Figure 3(a)). This increased expression was also detected at the protein level by an MBP ELISA in response to Fasudil and Y0503 when the cells were exposed to these inhibitors with periodic replacement of the compound (treatments at 24 hr and 72 hr after plating, Figure 3(c)).

**Figure 4.**
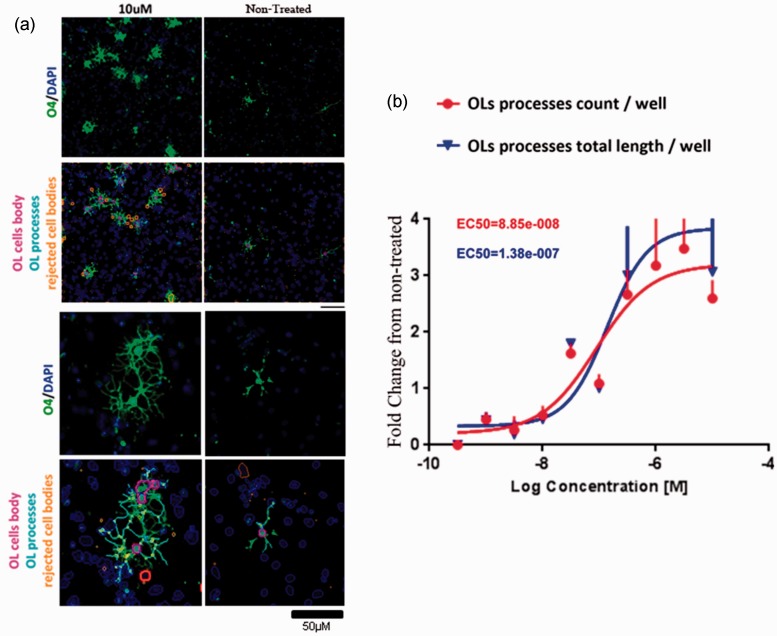
Human oligodendrocyte precursor cells (OPCs) differentiate in response to Rho-associated kinase (ROCK) inhibition. Human pluripotent stem cell-derived OPCs were treated with Y0503 for 48 hr. The media were then changed and subsequently renewed every other day for 21 days (see Materials and Methods section). At that time, immunostaining for the premyelinating OPC marker O4 was performed demonstrating a dose-response differentiation effect of ROCK inhibition (a, green: O4; blue: nuclei staining with 4′,6-diamidino-2-phenylindole, DAPI). Measurement of differentiation was performed by high content imaging and neurite outgrowth analysis using Cellomics’ ArrayScan device and software (b). Color-coded over-imposed images denote OPC cell bodies (pink), processes (cyan), rejected cell bodies representing dead cells or artifacts (orange), and nuclei (blue). NC: Negative control. Upper panels show images at low-power magnification (scale bar: 100 µM; lower scale bar: 50 µM).

We additionally studied the effect of ROCK inhibition in cultures of OPCs of human origin. These can be obtained from dissociated brain biopsies or embryonic CNS tissue (Monaco et al, 2012), but alternative methodologies to drive embryonic stem cells into the oligodendrocyte lineage have been described (Izrael et al., 2007; Li et al., 2013; Ogawa, Tokumoto, Miyake, & Nagamune, 2011). In this study, hSC-OPCs were utilized to analyze the effect of ROCK inhibition on OPC maturation in a protocol optimized to measure numbers of O4-positive cells after stimulus (see Materials and Methods section). Rock inhibitor was added once for the first 48 hr, and the total culture duration was 21 days. hSC-OPCs responded to ROCK inhibition in a similar fashion as cells of rodent origin showing morphological differentiation into the premyelinating stage (O4 positive) in a dose-response manner (Figure 4).

### Target Engagement and ROCK Isoform Definition in OPCs

**Figure 5.**
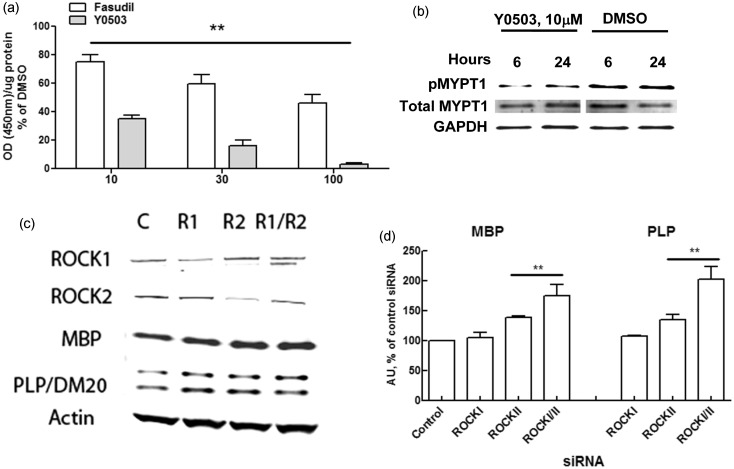
Compound efficacy and Rho-associated kinase (ROCK) isoform analysis. Fasudil and Y0503 efficiently enter the cell membrane and inhibit ROCK in oligodendrocyte precursor cells (OPCs) as determined by measurement of phosphorylation of ROCK’s downstream target myosin phosphatase subunit 1 (MYPT1) in mNph-OPC. Measurement of MYPT1 phosphorylation state by ELISA and western blot are shown in a and b, respectively. Inhibitory effect of Fasudil reaches 60% of control levels at 100 µM, while Y0503 decreased pMYPT1 near 80% at 30 µM and more than 80% at 100 µM in a 4-hr mNph-OPC treatment (a, data are presented as average ± SEM of three separate experiments performed in triplicate. **p < 0.01 according to Student *t* test). In cells exposed to Y0503 for 6 hr, downregulation of MYPT1 phosphorylation was maintained for over 12 hr, returning to control levels after 24 hr of treatment (b, western blot is representative of two runs in the same conditions). Small interfering RNA (siRNA)-mediated downregulation of ROCKI and ROCKII expression was used to gain insight on the specific relevance of either ROCK isoform in mNph-OPCs. Knockdown of either or both isoforms was up to ∼60% of noncoding control siRNA levels (c). However, a measurable increase in myelin basic protein (MBP) and proteolipid protein (PLP) expression was observed by western blot (c) when ROCKII was downregulated as compared with siRNA control (1.3-fold). Moreover, the increase in MBP expression was higher (∼2-fold) when the expression of both isoforms was decreased (band densitometry analysis in d of two experiments run in the same conditions). **p < 0.01 according to Student *t* test.

While commercially available ROCK inhibitors like Fasudil or Y0503 are widely used in basic research, their selectivity for ROCK versus other kinases has been shown to be low (Davies, Reddy, Caivano, & Cohen, 2000). Fasudil, for instance, is known to inhibit PKA and p70S6K with similar IC_50_ values as ROCKI and II (Breitenlechner et al., 2003; Tamura et al., 2005). Some of the overall effect of these inhibitors on oligodendrocyte differentiation may be contributed by these additional activities, making it harder to evaluate the differentiation-inducing effect of selectively inhibiting ROCK. In these circumstances, it is helpful to have a direct demonstration of target engagement by the inhibitor. Such assays are also important for establishing pharmacodynamic activity in in vivo models (Gabrielsson, Green, & Van der Graaf, 2010; Paweletz et al., 2011). Analyses of phosphorylation of cellular downstream targets of ROCK have been used in other studies to assess target engagement. A well-known marker of ROCK activity is myosin phosphatase subunit 1 (MYPT1), which dephosphorylates MLC inactivating it. Conversely, on phosphorylation by ROCK, MYPT1 is rendered inactive, therefore increasing MLC activity levels (Birukova et al., 2004; Tsai & Jiang, 2006). We assessed MYPT1 phosphorylation in Nph-OPCs treated for 4 hr with ROCK inhibitors in a dose-response experiment and using a sandwich ELISA as a measuring method (MBL International). Both Fasudil and Y0503 treatments reduced MYPT1 phosphorylation in OPCs as an indirect indication of ROCK inhibition by these compounds (Figure 5(a)). MYPT1 phosphorylation was also measured by western blot with similar results. In these experiments, we observed a peak of activity at 4 to 6 hr after treatment that was still detectable after 12 hr, reaching control levels by 24 to 30 hr in the presence of compound (Figure 5(b)).

**Figure 6.**
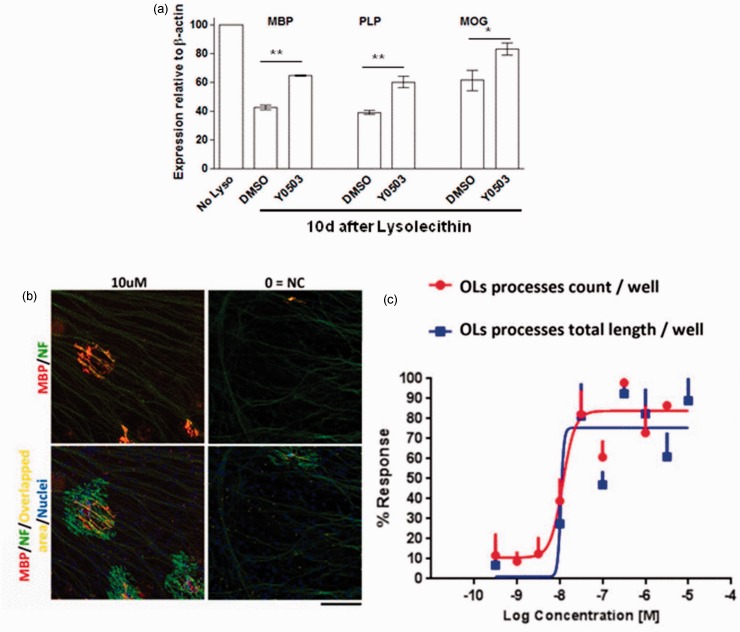
Oligodendrocyte precursor cell (OPC) differentiation induced by Rho-associated kinase (ROCK) inhibition results in enhanced myelination in two different in vitro models. Rat cerebellar slices maintained in vitro for 12 days were demyelinated with lysophosphatidylcholine (lysolecithin, 0.5 µg/ml, 17 hr) and allowed to spontaneously remyelinate during 10 additional days in the presence or absence (DMSO control) of Y0503 (10 µM). Treatment was renewed every 48 hr. Quantitative PCR for the myelin proteins myelin basic protein (MBP), proteolipid protein (PLP), and myelin oligodendrocyte glycoprotein (MOG) demonstrated an increased expression in slices exposed to ROCK inhibition as an indication of enhanced remyelination (a). In b, images of MBP-positive hPSC-OPCs (red) sending processes in alignment with neurofilament-stained axons (NF, green) generated from rat dorsal root ganglion neurons suggestive of myelination. Cocultures were grown for 28 days and treated with Y0503 for the first 48 hr. Image analysis of overlapping immunostaining was performed with neurite outgrowth measurement software resulting in a dose-response effect of ROCK inhibition in a number of oligodendrocyte processes over NF-positive axons (c). NC: Negative control. Scale bar: 200 µM.

ROCK is a serine/threonine kinase (see Amin et al., 2013 and Schofield & Bernard, 2013, for a review) of the PKA/PKC/PKG family of which there are two isoforms, ROCKI and ROCKII, sharing a high homology at both the amino acid level (∼76%) and in the kinase domain (∼97%; Nakagawa et al., 1996). Publically available ROCK inhibitors show poor selectivity between the two isoforms, making them unsuitable tools to investigate whether either isoform is more important for OPC differentiation. Fasudil, for example, presents an IC_50_ of 370 nM and 170 nM on ROCKI and ROCKII, respectively, in an enzymatic assay (EMD Millipore;unpublished data), whereas inhibitors such as Y0503 showed 60 nM and 20 nM, respectively. To approach this question, we utilized an siRNA approach to determine the effect of knocking down either isoform selectively. As is common with this technique, we were able to achieve only partial knockdown; the maximum reduction we achieved in cultured OPCs using five different siRNA sequences against ROCKI, ROCKII, or both was ∼60% relative to control noncoding siRNA (Figures 5 and 6). Although less than optimal knockdown was achieved, we were nevertheless able to measure a significant increase in MBP and PLP in cells with downregulated ROCKII. On the other hand, although knockdown of ROCKI alone did not result in any increase in myelin protein expression, we consistently saw a statistically significant increase in both MBP and PLP mRNA and protein in experiments in which both isoforms were knocked down simultaneously (Figure 5(c) and (d)). Taking into account the modest siRNA-mediated knockdown achieved in our assays, these results may indicate that either both isoforms are involved in OPC cytoskeletal organization or one may compensate for deficits in the other.

### OPC Differentiation Induced by ROCK Inhibition Results in Myelination of Adjacent Axons In Vitro

The experiments described thus far indicate that inhibition of ROCK results in OPC maturation as measured by morphology changes and increased myelin protein gene expression, but they do not address the final stage of differentiation, that is, myelination. We therefore tested the myelination potential of OPCs differentiated in response to ROCKi in two different in vitro systems: cerebellar organotypic cultures, and cocultures of hSC-OPCs and rat dorsal root ganglion (DRG) neurons. The first methodology allowed for the analysis of myelin protein reexpression in a myelination–demyelination–remyelination paradigm, and the second assay produced visual and measurable evidence of MBP/NF alignment as an indication of OPC–neuron interaction and myelination.

qPCR analysis of MBP, PLP, and MOG in lysolecithin-demyelinated cerebellar slices subsequently treated with Y0503 (one dose, 10 µM in a 10-day incubation post-lysolecithin) showed a significant increase in the expression of these myelin protein mRNAs as compared with slices treated with vehicle only (0.1% DMSO, Figure 6(a)). In these organotypic cultures, we observed apical areas of Purkinje cell axons aligned with MBP-positive membrane, which further indicated de novo formation of myelin (not shown).

Myelination in OPC-DRG cocultures is evidenced by alignment of OL extensions with axons, visualized by immunocytochemistry for MBP and NFs (Czepiel et al., 2011; Dincman, Beare, Ohri, & Whittemore, 2012). This methodology has been extensively characterized, and this alignment is widely accepted as an indication of myelination (Czepiel et al., 2011). We used hPSC-OPCs plated on 2-week-old DRG cultures cocultured for 28 days. Y0503 was given for the first 48 hr, after which media were changed every 48 hr. We observed an increased association of OPCs with neuronal fibers in a pattern indicative of axonal wrapping by OPC membranes forming myelin segments. Analysis of myelinated sections with a Cellomics neurite outgrowth module (Cellomics Technology, LLC, Rockville, MD) showed a dose-response effect on ROCK inhibition in terms of number and length of oligodendrocyte processes (MBP positive) colocalized with NF-positive axons (Figure 6(b) and (c)).

## Discussion

Current immunomodulatory therapies for MS are directed at peripheral elements of the immune system and have been successful in controlling relapses during the earlier relapsing–remitting phase. However, they do not prevent disease evolution to the progressive phase and appear to make no impact on the processes of demyelination, which lead eventually to shrinkage of the brain parenchyma and severe disability (Duddy et al., 2011; Nakahara, Maeda, Aiso, & Suzuki, 2012). The possibility of neuroprotective and remyelination-promoting therapies has attracted attention in recent years as therapeutic approaches that would complement existing anti-inflammatory therapies and which might impact the progressive phase of the disease (Stroet, Linker, & Gold, 2013; Zhornitsky et al., 2013). Remyelination has been shown to occur in early MS where active demyelinated lesions resolve into shadow plaques as observed by means of magnetic resonance imaging (MRI) in living patients and demonstrated in postmortem MS tissue by immunohistochemistry (Brown, Narayanan, & Arnold, 2012; Fox et al., 2011; Franklin, ffrench-Constant, Edgar, & Smith, 2012). However, remyelination diminishes as the disease progresses.

Conditions for an effective remyelination include the presence of OPCs in the lesions, a microenvironment permissive of OPC differentiation, and a suitable and receptive substrate for myelination, that is, the axons themselves. Indeed, there is a growing body of literature addressing the induction of OPC differentiation by stimulation or inhibition of a variety of cell signaling pathways (Deshmukh et al., 2013; Jepson et al., 2012; Medina-Rodriguez et al., 2013; Schmidt et al., 2012; Xiao, 2012; Zuchero & Barres, 2013). These signaling pathways include those leading to modifications of the cytoskeleton, and of these, the Rho kinase pathway is of special interest, as it appears to offer potential drug targets with the possibility of affecting the earliest stages of OPC maturation (Chang et al., 2009; Hahmann & Schroeter, 2010; Henderson et al., 2010; Olson, 2008).

Indirect and direct involvement of the Rho/ROCK signaling pathway in oligodendrocyte maturation and myelination has been previously reported by other authors. Paintlia et al. (2005, 2008), for instance, linked Rho/ROCK inhibition by statins to an increased OPC differentiation and survival in addition to a marked protection against demyelination in experimental allergic encephalomyelitis models. In an in vitro study, the regulation of oligodendrocyte process dynamics and survival by simvastatin was characterized in cultured cells as a function of ROCK inhibition (Miron et al., 2007). Furthermore, it has been reported using an in vitro spinal cord injury model containing rat Nph-derived astrocytes in coculture with spinal cord cell suspensions that exposure to Rho/ROCK inhibition enhanced neurite outgrowth and myelination of previously scraped cell layers (Boomkamp, Riehle, Wood, Olson, & Barnett, 2012).

Although these studies indicate an active involvement of the Rho/ROCK signaling pathway in OPC differentiation and myelin dynamics, how ROCK regulates process extension and axonal wrapping is still incompletely understood. ROCK in its active form induces stress fiber formation and contraction in various cell types (for a review, see Chrzanowska-Wodnicka & Burridge, 1996; Tojkander, Gateva, & Lappalainen, 2012). A possible mechanism directing the differentiation response might be regulated by activation of downstream signaling molecules in OPCs, including phosphorylation of MLC, which is directly implicated in the formation of stress fibers by polymerization of actin and tubulin subunits. By inhibiting ROCK, phosphorylation of MLC is decreased, altering the generation of stress fibers. Stress fibers in turn modulate cell motility and spreading. In OPCs, inhibition of stress fiber formation and actin polymerization results in increased process extension and differentiation conducive to myelination. It appears that this mechanism affects both early OPC differentiation and axonal wrapping and has been elegantly described by Wang et al. (2008) and Wang et al. (2012). Interestingly, the state of MLC required to promote OPC differentiation differs from that required for myelination of peripheral nerves by Schwann cells, where it must be active to induce axonal wrapping (Kippert, Fitzner, Helenius, & Simons, 2009; Wang et al., 2008) and where ROCK inhibition has no enhancing effects on the membrane extension of these cells (Melendez-Vasquez, Einheber, & Salzer, 2004).

In our experimentation with OPCs, we did not observe significant changes in cell proliferation induced by ROCK inhibition. This is of relevance, as direct blockage of cell cycle in OPCs would invariably result in cell differentiation (Dugas, Ibrahim, & Barres, 2007). Correspondingly, high ROCK activity has been closely related to cell proliferation in cancer cells where ROCK inhibition has been shown to have antimitogenic effects (L. Chen, Qu, et al., 2013; Zhang et al., 2013).

Here, we describe the effect of ROCK inhibition on OPC differentiation and induction of myelination using well-characterized pharmacological tool compounds with specificity for this kinase (M. Chen, Liu, et al., 2013; Liao, Seto, & Noma, 2007; Mueller, Mack, & Teusch, 2005; Raad et al., 2012). We observed and quantitated stimulation of OPC differentiation in response to these reagents, which produced a massive extension of branches and processes, followed by an increased and sustained expression of myelin components, which was followed in turn by the formation of myelin in the presence of axons in our organotypic myelinating cultures and human OPC-DRG cocultures. This is a critical observation, as it indicates that the differentiation initiated by ROCK inhibition proceeds to completion in the presence of axons and does not halt at an earlier stage. We observed induction of differentiation by ROCKi in cells of mouse, rat, and human origin, of differing derivation and stage of maturation, thus validating the relevance of the observations across species. Not only did OPC of human origin respond to ROCK inhibition, but in addition, myelin formation was also observed as alignment of MBP-positive extensions overlapping with NF-positive fibers. These findings indicate that the observations implicating ROCK inhibition as a mechanism for promoting OPC differentiation and myelination have therapeutic implications.

An unexpected conclusion from the work described here is that both ROCK isoforms appear to play a role in the promotion of OPC differentiation. In studies using knockout mice, it has been shown that ROCKI and ROCKII phosphorylate the same downstream substrates resulting in no discernible differences in the biological effects (Thumkeo, Shimizu, Sakamoto, Yamada, & Narumiya, 2005). However, cell- or tissue-type expression of one or another isoform might in some cases lead to a selective involvement of that isoform in tissue-specific effects. Thus, some reports claim a specific role of ROCKI in the control of cardiovascular cytoskeletal events (Jiang et al., 2007; Rikitake et al., 2005) and of ROCKII in the control of immune responses in EAE (Biswas et al., 2010; Sun et al., 2006; Yu et al., 2010). We have shown that OPCs express both isoforms, and although using siRNA we were not able to inactivate completely either of the isoforms selectively, we did observe an increased level of myelin protein expression when both isoforms were ∼55% to 65% downregulated. The high homology at the amino acid level (76%) and kinase domain (97%) between the two isoforms, together with similar levels of expression in the CNS in general and specifically in OPCs, indicate a likely similarity in biological functions or probable compensatory mechanisms when one of the isoforms is absent. Thus, a dual ROCKI/ROCKII inhibitor is likely to achieve greater efficacy in modulating the OPC cytoskeleton and subsequently promoting differentiation than an isoform-selective inhibitor.

Our research addresses the role of ROCK in the induction of oligodendrocyte differentiation and formation of myelin and suggests that it is a potential therapeutic target for the development of remyelination-promoting therapy. In the specific case of MS, it has additional attraction, as ROCK inhibition has been reported to downregulate the exacerbated immune response in the EAE model of MS, which would provide an added benefit to a remyelination therapy (Sun et al., 2006; Yu et al., 2010). At present, tool compounds with the appropriate combination of oral availability, brain penetration, and selectivity against other kinases are not yet available for testing in in vivo models of demyelination–remyelination. In addition to efficacy, safety is a key consideration in drug development. The major possible liabilities of ROCK as a therapeutic target stem from possible cardiovascular effects (Duong-Quy, Beim, Lium, & Dinh-Xuanm, 2013; Ryan et al., 2013; Sehon et al., 2008; Shi & Wei, 2013) and possible side effects related to alterations in glucose transport (Chun et al., 2012; Ishikura, Koshkina, & Klip, 2008). However, it is encouraging that Fasudil, a relatively weak ROCK inhibitor (IC_50_ in enzymatic assays: ROCKI 370 nM and ROCKII 170 nM), has been in clinical use for the treatment of cerebral vasospasm in Japan with no reported adverse side effects (Dong, Yan, & Yu, 2009; Satoh et al., 2012).

## Summary

Oligodendrocyte differentiation and myelin regeneration have become the focus of new therapy development for demyelinating diseases. We demonstrate that exposure of oligodendrocyte progenitors to inhibitors of Rho-associated kinase results in cell maturation and myelin formation.
